# The Osaka prognostic score and Naples prognostic score: novel biomarkers for predicting short-term outcomes after spontaneous intracerebral hemorrhage

**DOI:** 10.1186/s12883-023-03287-3

**Published:** 2023-07-18

**Authors:** Rui Liu, Changcun Chen, Yutong Zhao, Yuguang Tang, Weiwei Shen, Zongyi Xie

**Affiliations:** 1 Department of Neurosurgery, The Second Affiliated Hospital, Chongqing Medical University, 76 Linjiang Road, Chongqing, 400010 People’s Republic of China; 2 Department of Endocrinology, The First Affiliated Hospital, Chongqing Medical and Pharmaceutical College, 301 Dashi Road,, 400060 Chongqing, People’s Republic of China

**Keywords:** Intracerebral hemorrhage (ICH), Outcome, Osaka prognostic score (OPS), Naples prognostic score (NPS)

## Abstract

**Objectives:**

Poor immune-nutritional status has been associated with an unfavorable outcome in critical illness. The Osaka prognostic score (OPS) and the Naples prognostic score (NPS), based on inflammatory and nutritional status, has been shown to predict prognosis following cancer and other diseases. The aim of this study was to investigate the relationship between the OPS and NPS and the short-term outcomes of patients with intracerebral hemorrhage (ICH).

**Methods:**

We retrospectively analyzed the clinical data of patients hospitalized with spontaneous ICH (*n* = 340) at The Second Affiliated Hospital of Chongqing Medical University between August 2016 and August 2021. Inclusion criteria included patients aged between 18 and 70, and if a blood sample was taken for laboratory testing within 24 h of admission (serum C-reactive protein, albumin, total cholesterol, and counts for neutrophils, lymphocytes, and monocytes were collected on admission). Exclusion criteria included a non-spontaneous cause of ICH and patient death during hospitalization. Patients were divided into four groups based on OPS or five groups according to NPS. Outcomes were evaluated by the modified Rankin Scale (mRS) at six months post-ICH hospitalization. An unfavorable outcome was defined as a mRS score ≥ 3.

**Results:**

A total of 289 patients met our inclusion criteria. The unfavorable outcome group had older age, a lower Glasgow Coma Scale score, a higher rate of complications and cerebral herniation, a longer hospital stay, and higher OPS and NPS when compared with the favorable outcome group. Univariate analysis showed that both OPS and NPS were strongly correlated with mRS (*r* = 0.196,*P* < 0.001; *r* = 0.244, *P* = 0.001, respectively). Multivariate analysis further showed that OPS and NPS were both independent predictors of unfavorable outcomes for patients with ICH with adjusted odds ratios of 1.802 (95% confidence interval [CI]:1.140–2.847, *P* = 0.012) and 1.702 (95% CI: 1.225–2.635, *P* = 0.02), respectively. The area under the curve (AUC) of NPS for predicting a poor outcome was 0.732 (95% CI: 0.665–0.799), which was similar to the AUC of OPS 0.724 (95% CI: 0.657–0.792).

**Conclusions:**

In this cohort, a higher OPS and NPS on admission was associated with poor outcome at six months following ICH, supporting their potential role as markers for predicting the outcome of patients with ICH.

## Introduction

Intracerebral hemorrhage (ICH) accounts for approximately 10 to 15% of all stroke cases and has a high mortality rate [1, 2]. The clinical outcomes of patients with ICH are known to be influenced by a variety of factors, including age, hematoma volume and location, peri-hemorrhagic edema, intracranial pressure, neurological impairment, inflammatory response, and nutritional status [3–5]. A mounting body of preclinical evidence has shown that a marked inflammatory response and malnutrition are associated with an unfavorable outcome after ICH [6, 7]. Previous studies have reported that various indicators can predict the outcomes of patients with ICH [8–10]; however, few of these involve specific blood biochemical tests. Furthermore, a variety of scoring systems have also been proposed to estimate the prognosis of patients, including the controlling nutritional status (CONUT) score, the prognostic nutrition index (PNI) and the systemic inflammation score (SIS) [11–13]. However, these scoring systems tend to focus solely on inflammation or nutritional levels and therefore cannot accurately estimate outcomes. As such, there is need for a novel scoring system which combines inflammation and nutritional status with predictive value for the prognosis of patients with ICH to help clinicians efficiently stratify patients for individualized treatment modalities.

Recently, the Osaka prognostic score (OPS) and the Naples prognostic score (NPS) have been reported as objective and easily applicable biomarkers for predicting the prognosis of patients with cancer [14–16]. The OPS, a new prognostic score incorporating C-reactive protein (CRP), serum albumin and total lymphocyte counts (TLC), was first used to predict outcome for colorectal cancer following resection [14]. The NPS is also a new scoring system that includes serum albumin, total cholesterol (TC) concentration, neutrophil-lymphocyte ratio (NLR) and lymphocyte-monocyte ratio (LMR), and can comprehensively reflect a patient’s immune and nutritional status. The NPS was first used in patients with colorectal cancer and was found to be an independent prognostic indicator for patients undergoing surgery for colorectal cancer [17]. However, it is unclear whether the OPS and NPS could be used as viable biomarkers for predicting the outcomes of patients with ICH.

The aim of the present study was to investigate the relationship between OPS and NPS and the short-term outcome of patients with ICH.

## Materials and methods

### Patients

We conducted a retrospective analysis of a database of consecutive patients diagnosed with spontaneous ICH admitted to The Second Affiliated Hospital of Chongqing Medical University between the 1st of August 2016 and the 31st of August 2021. A total of 340 patients were identified. Patients were included if ICH was spontaneous, if they were aged between 18 and 70, and if a blood sample was taken for laboratory testing within 24 h of admission. Exclusion criteria included: (1) a non-spontaneous cause of ICH (including traumatic ICH, metastatic hemorrhagic cerebral lesions, ICH related to brain tumor, hemorrhagic transformation of cerebral infarction, vascular abnormality, or any other suspected secondary causes); (2) if ICH was attributable to a supra-therapeutic international normalized ratio in the setting of prehospital anticoagulation or coagulopathy, and primary intraventricular hemorrhage (IVH); (3) if there was an active infection or autoimmune disease, and; (4) if the patient died during hospitalization.

This study was approved by the Ethics Committee of The Second Affiliated Hospital of Chongqing Medical University(NO.202,169).

### Data acquisition

A range of clinical and laboratory variables were acquired from the electronic medical record (EMR) system. Patient demographics (i.e., age, gender, temperature, and pulse rate), ICH hematoma volume and location were recorded upon admission [3]. The ICH volume was measured using the ABC/2 method, where A means the greatest hemorrhage diameter by computed tomography (CT), B means the diameter 90° to A, and C is the approximate number of CT slices with hemorrhage multiplied by the slice thickness. Blood samples were obtained for laboratory testing within 24 h of admission and stroke status was evaluated by experienced nurses during the period of hospitalization. A range of baseline vascular risk factors were also recorded, including stroke history, hypertension, diabetes mellitus, dyslipidemia, atrial fibrillation (a history of atrial fibrillation was based on the confirmation of at least one electrocardiogram, or the onset of arrhythmia during hospitalization), cardiovascular disease, smoking, and alcohol consumption. Other risk factors, such as cancer and lung disease, were also recorded, as well as cerebral herniation and surgery performed during the period of hospitalization. Medical complications, including infection, epilepsy and deep vein thrombosis were also recorded during the period of hospitalization. We used the ICH score and Glasgow Coma Scale (GCS) to assess the severity of neurological impairment within 24 h of patient admission [8]. The GCS is a tool used to aid in objectively measuring the neurological status of a patient and consists of three aspects: eye opening response, verbal response, and motor response. We defined patients with an ICH score of 0–1 as the mild neurological impairment group and those with an ICH score of 2–4 as a more severe neurological impairment group. The ICH score is a simple clinical grading scale that allows risk stratification on presentation with ICH. The ICH score was the sum of individual points assigned as follows: GCS score 3 to 4 (= 2 points), 5 to 12 (= 1 point), 13 to 15 (= 0 point); age > = 80 years yes (= 1 point), no (= 0 point); infratentorial origin *yes* (= 1point), *no* (= 0 point); ICH volume > = 30 cm3 (= 1 point), < 30 cm3 (= 0 point); and intraventricular hemorrhage *yes* (= 1 point), *no* (= 0 point). Routine laboratory tests data were also recorded.

For the OPS, the cut-off values were set as > 6.5 mg/dL for CRP, < 4.4 g/dl for serum albumin, and < 1015 for TLC. Each index was used only once and allocated a single point [18]. Next, the points were added together and the patients were divided into four groups according to the total score (zero, one, two or three points). The cut-off values were chosen from a receiver operating characteristic (ROC) curve for the modified Rankin Scale (mRS) of all patients. The NPS was based on four parameters: serum albumin, TC, LMR and NLR. According to the abovementioned ROC analysis, the cut-off values were set as serum albumin ≥ 4 g/dL, TC > 180 mg/dL, NLR < 2.96, LMR > 4.44, and was scored as 0, while serum albumin < 4 mg/dL, TC ≤ 180 mg/dL, NLR ≥ 2.96 and LMR ≤ 4.44 was scored as 1. The NPS was the sum of scores for each of the four parts [17].

To acquire information relating to the mRS and measure the functional recovery of patients after stroke, individual patients were evaluated *via* telephone follow-up at six months post-hospital discharge using a standardized interview protocol that was administered by trained interviewers. Patients were divided into a favorable outcome group with a low mRS score (< 3) and an unfavorable outcome group with a high mRS score (≥ 3). Patients who were lost to follow-up were assigned to the poor prognosis group.

### Statistical analyses

The analysis was performed with SPSS V23 and MedCalc Statistical Software version 19.0.7. For descriptive analysis, we used frequency and percentages for categorical variables and mean with standard deviation (SD) for continuous variables. To compare categorical variables, we used the Chi-squared test or Fisher’s exact test. For continuous variables, we used the Mann-Whitney U test or unpaired t-test. Associations between OPS, NPS and adverse outcomes were analyzed by **univariate and multivariate logistic regression**. The odds ratio (OR) and 95% confidence interval (CI) of each factor was also calculated. The ROC analysis was performed to evaluate associations between the performance of each predictive score on admission and the 6-month clinical outcomes. To determine the optimal cut-off value of OPS and NPS components for predicting disease outcomes, ROC analyses were performed. According to the maximum value of the Youden index (sensitivity + specificity − 1), the optimal critical point was obtained. To assess the importance of OPS and NPS in the clinical context, validated clinical grading scales, such as CONUT score, PNI and SIS, were included in the abovementioned ROC analysis. The area under the curve (AUCs) were **compared by the DeLong test.** All P values were two-sided, and significance was set at *P* < 0.05.

## Results

### Patient characteristics

The data from 289 patients was included in the analysis and 11 (3.8%) patients were lost to follow-up. Baseline characteristics for the patients are presented in Table 1. Of the 289 patients, 175 (60.6%) had a favorable outcome (mRS < 3) at 6 months and 114 (39.4%) patients had a poor outcome. Patients were divided into four OPS groups(0, 1, 2 and 3) and five NPS groups (0, 1, 2, 3 and 4). There were no significant differences in clinical characteristics between four OPS groups and five NPS groups. In the favorable outcome group, the mean age was 54 years (interquartile range, 48–61 years), 120 (68.6%) patients were male and the median ICH score was 1 point [interquartile range, 0–4 points]. The mean hospital stay was 13 days (interquartile range, 9–17 days) and the median GCS was 15 (interquartile range, 13–15). Twenty-two (12.6%) patients underwent surgery after admission. In the poor outcome group, the mean age was 58 years (interquartile range, 50.75–64 years), 75 (65.8%) patients were male and the median ICH score was 1 point [interquartile range, 0–4 points]. The mean hospital stay was 17.5 days (interquartile range, 13.75–25 days) and the median GCS was 13 (interquartile range, 9–15). Forty-seven (41.2%) patients underwent surgery after admission. Patients in the poor outcome group were more likely to have complications than the favorable outcome group (*P* < 0.001, Table 1).

**Table 1:** Characteristics of patients with spontaneous intracerebral hemorrhage

Characteristics	Favorable outcome(*n* = 175, 0–2)	Poor outcome (*n* = 114, 3–6)	*P* value
Age(years)	54.00	58.00	0.013^c^
Mean (SD)	(48.00–61.00)	(50.75-64.00)	
Gender			
Male	120(68.6%)	75(65.6%)	0.622^b^
Female	55(31.4%)	39(34.4%)	
ICH score			
0(0–1)	152(86.9%)	64(56.1%)	< 0.001^b^
1(2–4)	23(13.1%)	50(43.9%)	
Cerebral hernia	2(1.1%)	10(8.8%)	0.004^a^
Hematoma expansion	10(5.7%)	14(12.3%)	0.048^a^
Intraventricular hemorrhage	49(28.0%)	45(39.5%)	0.042^b^
Surgery	22(12.6%)	47(41.2%)	< 0.001^b^
Complication			
infection	35(20.0%)	50(43.9%)	< 0.001^b^
epilepsy	5(2.9%)	6(5.3%)	
deep vein thrombosis	7(4.0%)	6(5.3%)	
hydrocephalus	6(3.4%)	4(3.5%)	
Hypertension	144(82.3%)	92(80.7%)	0.734^b^
Diabetes	18(10.3%)	11(9.6%)	0.860^a^
Cerebral infarction	6(3.4%)	6(5.3%)	0.445^a^
Cerebral hemorrhage	4(2.2%)	5(4.4%)	0.315^a^
Smoking	72(41.1%)	52(45.6%)	0.453^b^
Drinking	50(28.6%)	37(32.5%)	0.482^b^
Basic diseases			
cancer	25(14.3%)	10(8.8%)	0.310^b^
lung diseases	24(13.7%)	8(7.0%)	
other diseases	6(3.4%)	6(5.3%)	
GCS	15.00(13.00–15.00)	13.00(9.00–15.00)	< 0.001^c^
Hospital stays(day)	13.00(9.00–17.00)	17.5(13.75-25.00)	< 0.001^d^
Albumin(g/dL)	4.24(3.98–4.50)	4.21(3.91–4.45)	0.249^c^
Total cholesterol(mg/dL)	4.62(4.00-5.29)	4.52(3.85–5.06)	0.061^c^
C-reactive protein(mg/dL)	2.03(0.79–4.08)	2.14(0.85–7.39)	0.191^c^
Monocytes(10^9/L)	0.40(0.29–0.57)	0.45(0.31–0.70)	0.074^c^
Lymphocyte(10^9/L)	1.17(0.92–1.52)	1.00(0.72–1.36)	0.002^c^
Neutrophils(10^9/L)	6.81(5.03–9.03)	8.35(6.03–11.13)	0.001^c^
Hemoglobin(g/L)	142.00(133.00-154.00)	137.50(130.00-150.00)	0.085^c^
Platelet(10^9/L)	196.00(153.00-239.00)	192.50(145.75-239.25)	0.453^c^
COUNT			
1	56(32.0%)	26(22.8%)	0.019^d^
2	109(62.3%)	75(65.8%)	
3	10(5.7%)	11(9.6%)	
4	0(0.0%)	2(1.8%)	
SIS			
0	25(14.3%)	7(6.1%)	0.017^d^
1	119(68.0%)	74(64.9%)	
2	31(17.7%)	33(29.0%)	
PNI			
0	77(44.0%)	72(63.2%)	0.001^d^
1	98(56.0%)	42(36.8%)	
LMR	2.97(2.05–4.22)	2.28(1.50–3.37)	< 0.001^c^
OPS			
0	0(0.0%)	0(0.0%)	< 0.001^d^
1	53(30.3%)	26(22.8%)	
2	110(62.9%)	60(52.6%)	
3	12(6.8%)	28(34.4%)	
NPS			
0	4(2.3%)	0(0.0%)	< 0.001^d^
1	21(12%)	4(3.5%)	
2	76(43.4%)	35(30.7%)	
3	54(30.9%)	53(46.5%)	
4	20(11.4%)	22(19.3%)	

*Abbreviations*: *GCS *Glasgow coma scale, *NLR *Neutrophil-lymphocyte ratio, *LMR *Lymphocyte-monocyte ratio, *OPS *Osaka prognostic score, *NPS *Naples prognostic score

^a^Fisher’s exact test

^b^χ2 test

^c^ *t*-test /*U*-test

^d^Rank sum test

### OPS and NPS were strongly correlated with the mRS score

There were 79 patients (27.3%) in the OPS 1 group; of these, 53 patients (67.1%) had a favorable outcome; Compared to 170 patients (58.8%) in the OPS 2 group of which 110 patients (64.7%) had a favorable outcome. Of the 40 patients (13.8%) in the OPS 3 group, 30% had a good prognosis. The favorable prognosis rate at six months after discharge in the NPS groups 0, 1, 2 3 and 4 were 100%, 84.0%, 68.5%, 50.5% and 47.6%, respectively. Pearson correlation analysis showed that both OPS and NPS were strongly correlated with mRS (*r* = 0.196, *P* < 0.001; *r* = 0.244, *P* = 0.001, respectively) (Tables 2 and 3).

**Table 2:** Univariate analysis of the included patients according to 6-month prognosis outcome

Characteristics	OR	95% CI	*P* value
Age	1.026	1.001–1.052	0.043
Gender	0.881	0.534–1.455	0.622
ICH score	2.338	1.750–3.123	< 0.001
Cerebral hernia	8.317	1.787–38.701	0.007
Hematoma expansion	2.310	0.989–5.397	0.053
Intraventricular hemorrhage	1.677	1.017–2.765	0.043
Surgery	4.879	2.726–8.731	< 0.001
Complication	3.285	1.994–5.421	< 0.001
NLR	1.109	1.056–1.165	0.001
LMR	0.751	0.635–0.889	< 0.001
Hypertension	0.900	0.491–1.650	0.734
Diabetes	0.931	0.423–2.053	0.860
Cerebral infarction	1.565	0.492–4.977	0.448
Cerebral hemorrhage	1.961	0.515–7.463	0.323
Smoking	1.200	0.745–1.931	0.453
Drinking	1.201	0.721–2.003	0.482
Basic diseases	0.748	0.426–1.312	0.311
GCS	0.741	0.668–0.882	< 0.001
Hospital stays	1.077	1.043–1.112	< 0.001
Albumin	0.621	0.352–1.096	0.101
Total cholesterol	0.768	0.600-0.984	0.037
C-reactive protein	1.010	0.999–1.021	0.069
Monocytes	2.476	0.941–6.515	0.066
Lymphocyte	0.491	0.303–0.795	0.004
Neutrophils	1.102	1.035–1.175	0.003
Hemoglobin	0.990	0.976–1.004	0.154
Platelet	0.998	0.995–1.002	0.373
COUNT	1.632	1.080–2.466	0.020
SIS	1.864	1.207–2.878	0.005
PNI	0.458	0.283–0.743	0.003
OPS	1.933	1.303–2.867	< 0.001
NPS	1.818	1.360–2.430	0.001

*Abbreviations: GCS *Glasgow coma scale, *NLR *Neutrophil-lymphocyte ratio, *LMR *Lymphocyte-monocyte ratio, *OPS *Osaka prognostic score, *NPS *Naples prognostic score, *OR *Odds ratios, *95%CI *95% confidence interval

**Table 3:** Patient distribution in OPS and NPS

	Favorable outcome	Poor outcome	
OPS			
0	0	0	0
1	53(67.1%)	26(32.9%)	79(100%)
2	110(64.7)	60(35.3%)	170(100%)
3	12(30.0%)	28(70.0%)	40(100%)
NPS			
0	4(100%)	0	4(100%)
1	21(84%)	4(16%)	25(100%)
2	76(68.5%)	35(31.5%)	111(100%)
3	54(50.5%)	53(49.5%)	107(100%)
4	20(47.6%)	22(52.4%)	42(100%)

*Abbreviations: OPS *Osaka prognostic score, *NPS *Naples prognostic score;

### OPS and NPS were independent predictors for poor outcome in patients with ICH

When compared with the poor outcome group, more patients with a lower OPS and a lower NPS (*P* < 0.001) were observed in the favorable outcome group. Next, we applied univariate analysis to investigate the relationship between the 6-month outcome and clinical characteristics and found that patients in the poor outcome group were significantly older, had a lower GCS, a higher rate of complications and cerebral herniation, and a longer hospital stay than those in the favorable outcome group. Multivariate analysis of unfavorable outcomes led to the establishment of two models by multiple regression (OPS or NPS was included in both models). Multivariate analysis showed that both OPS and NPS were independent predictors for an unfavorable outcome in patients with ICH (Table 4); the adjusted odds ratio was 1.802 (95% CI:1.140–2.847, *P* = 0 0.012) and 1.702 (95% CI: 1.225–2.635, *P* = 0.02), respectively.

**Table 4:** Multivariate analyses of the involvement of different variables in patients with spontaneous intracerebral hemorrhage and poor functional outcome at 6-month follow-up Model 1 OPS

Characteristics	OR	95% CI	*P* Value
Model 1 OPS			
Age	1.026	0.996–1.056	0.093
Cerebral hernia	1.287	0.214–7.758	0.783
Surgery	0.515	0.245–1.802	0.080
Complication	0.878	0.453–1.703	0.701
GCS	0.910	0.783–1.058	0.220
Hospital stays	1.029	0.995–1.065	0.093
OPS	1.802	1.140–2.847	0.012
Constant	0.100		0.182
Model 2 NPS			
Age	1.031	1.001-1.062	0.039
Cerebral hernia	1.407	0.238-8.331	0.706
Surgery	0.534	0.254-1.126	0.099
Complication	0.822	0.424-1.592	0.561
GCS	0.896	0.770-1.042	0.154
Hospital stays	1.024	0.989-1.059	0.183
NPS	1.702	1.225-2.365	0.002
Constant	0.073		0.138

*Abbreviations*: *GCS *Glasgow coma scale, *OPS *Osaka prognostic score, *NPS* Naples prognostic score, *OR *Odds ratios, *95%CI *95% confidence interval

### The predictive value of OPS and NPS were superior to other scoring systems

**Fig. 1:**
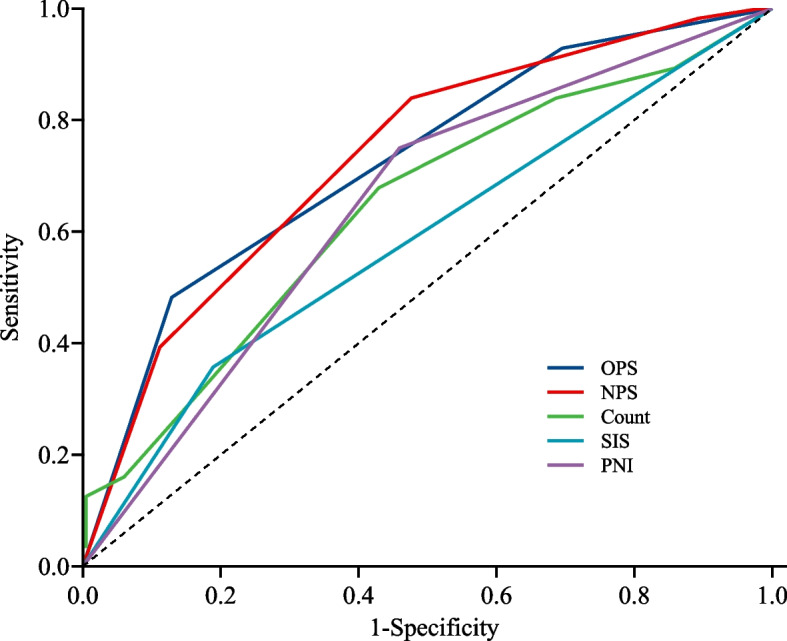
Receiver operating characteristic analysis of OPS, NPS, COUNT, SIS and PNI in predicting functional outcome of ICH. Abbreviations : OPS: Osaka prognostic score; NPS: Naples prognostic score; CONUT: controlling nutritional status; SIS: systemic inflammation score; PNI: prognostic nutrition index

The ROC curve for poor outcome showed that OPS and NPS had good predictive values. In the case of AUC > 0.5, the closer the AUC is to 1, the better the predictive values is. The AUC of NPS and OPS for predicting a poor outcome was 0.732 (95% CI: 0.665–0.799) and 0.724 (95% CI: 0.657–0.792) (Fig. 1), and the cut-off values for OPS and NPS were both 3. The sensitivity and specificity of the expected performance of the OPS were 48.2% and 87.1%, while those of NPS were 83.9% and 52.4%, respectively. The AUC for OPS and NPS were not significantly different, as determined by the DeLong method (*P* = 0.859) (Table 5). *P* > 0.05 show that there is no significant difference in the AUC of OPS and NPS.

**Table 5:** DeLong method compares the AUC of different systems

Characteristics		*P* Value
OPS	NPS	0.859
OPS	COUNT	0.027
OPS	SIS	0.01
OPS	PNI	0.037
OPS	LMR	0.044
OPS	NLR	0.043
NPS	COUNT	0.032
NPS	SIS	< 0.001
NPS	PNI	0.035
NPS	LMR	0.049
NPS	NLR	0.042

*Abbreviations*: *OPS *Osaka prognostic score, *NPS *Naples prognostic score, *CONUT *Controlling nutritional status, *SIS *Systemic inflammation score, *PNI *Prognostic nutrition index, *NLR *Neutrophil-lymphocyte ratio, *LMR *Lymphocyte-monocyte ratio

**Fig. 2:**
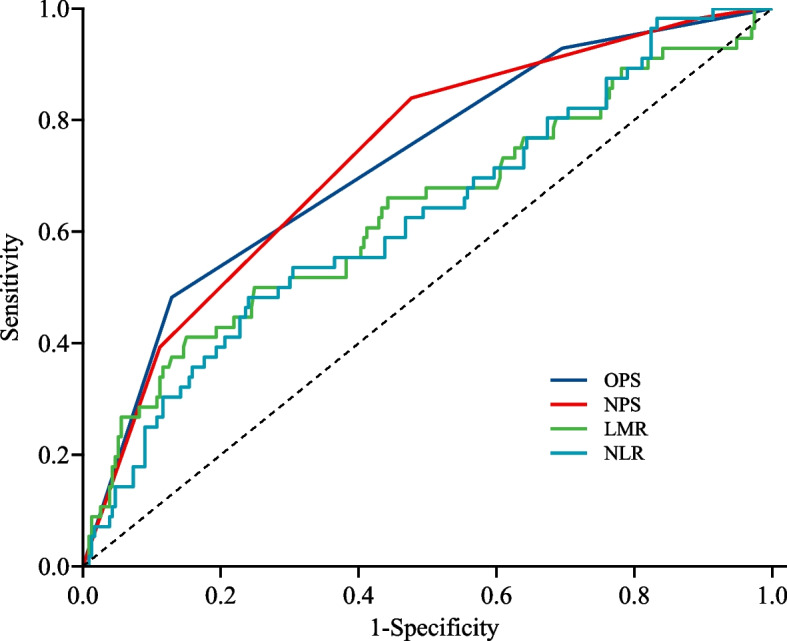
Receiver operating characteristic analysis of OPS, NPS, NLR and LMR in predicting functional outcome of ICH. Abbreviations : OPS: Osaka prognostic score; NPS: Naples prognostic score; NLR: neutrophil-lymphocyte ratio; LMR: lymphocyte-monocyte ratio

A comparison of the AUC of OPS, NPS, COUNT, SIS and PNI was performed. The AUC of COUNT, SIS and PNI for predicting outcomes at 6-months were 0.641 (95% CI 0.559–0.723), 0.585(95% CI 0.510–0.661), and 0.645 (95% CI 0.580–0.711), respectively. The AUC of OPS and NPS were significantly higher than those of the other scoring systems. A comparison of the AUC for these scoring systems (COUNT, SIS and PNI) and OPS and NPS by the DeLong method showed significant differences (Fig. 2 and Table 5).

## Discussion

To our knowledge, this is the first study to investigate the relationship between OPS, NPS and the short-term outcome of patients with spontaneous ICH. Our findings suggest that patients with a lower OPS and a lower NPS on admission had a more favorable prognosis than those with a higher OPS and a higher NPS at 6 months after ICH. The predictive value of OPS and NPS was not significantly different. Thus, OPS and NPS might be important independent predictors for the outcomes of patients with spontaneous ICH on admission.

There was evidence that inflammatory and nutritional indicators calculated based on routine blood parameters at admission, such as neutrophils, lymphocytes, serum albumin and total cholesterol, can provide valuable prognostic information for various diseases, including ICH and ischemic stroke [19–22]. In patients with ICH, inflammation occurs immediately after the formation of hematoma, and an increasing body of evidence shows that inflammation is one of the important factors underlying secondary brain injury after ICH [4, 23]. Since the inflammatory response can induce brain injury after ICH resulting in the loss of neurological function, anti-inflammatory strategies might represent potential treatments for patients with ICH. Metabolic demands and malnutrition frequently occur in ICH patients as a result of a highly stressed state, a disturbance of consciousness, and dysphagia [24]. Furthermore, malnutrition is associated with impaired immunological function and leads to increased rates of infection [7]. Thus, malnutrition and inflammation are inextricably connected. Our present results also showed that the group of ICH patients with a poor outcome group had a worse nutritional and inflammatory status.

The inflammatory immune response is involved in multiple pathological processes after ICH, including brain edema, neuroinflammation and neuronal damage [25]. A growing body of evidence suggests that stroke induces systemic immune dysregulation. During the acute phase after ICH, the sympathetic system and the hypothalamic-pituitary-adrenal axis become overactivated and the levels of catecholamines and steroids increase, thus contributing to systemic immunosuppression and further inducing the functional inactivation and apoptosis of peripheral lymphocytes [26]. Moreover, the lymphopenia in experimental models and patients after ICH also suggests that intracerebral hemorrhage may impair peripheral immunity [27].

Lymphocytes play a crucial role in immune regulation and host defense against pathogens, and their count is an indicator of immune status. Post-stroke immunosuppression, often characterized by lymphopenia upon stroke presentation, decreases immune capacity as lymphocyte numbers reduce, which increases the risk of infection after ICH and may have an impact on functional outcomes [28, 29]. Our findings suggest that ICH patients with lower lymphocyte counts are associated with poor outcomes.

In addition, a higher NLR and a lower LMR have been shown to be associated with increased mortality and poor functional outcome across a range of diseases [30, 31]. Previous studies have suggested a higher NLR was associated with worse outcome after stroke [30, 32, 33]. Two of the largest prospective studies both showed that a higher NLR was associated with a worse initial neurological status and a larger hematoma volume on hospital admission [30]. In ischemic stroke, a higher NLR is also an independent predictor for mortality at six months [34]. Furthermore, two separate prospective clinical studies on ICH reported that an increased monocyte count on presentation of ICH was associated with poor outcomes and higher mortality [35, 36]. A higher monocyte count or reduced LMR has also been linked to poor outcome in ischemic stroke [31]. Consistent with previous findings, our results showed that a higher NLR and lower LMR were associated with poor prognosis in ICH patients. However, the prognosis of patients with ICH is not only related to the levels of inflammation, but also to nutritional status. The OPS and NPS combined with inflammatory and nutritional status were shown to predict outcomes in a more comprehensive manner [14–16]. Our results have shown that OPS and NPS have better predictive value than LMR or NLR alone. The combination of additional indicators of nutritional levels may result in a better stratification for ICH patients in terms of prognosis than NLR and LMR. Furthermore, we have shown that OPS and NPS can better predict the prognosis of patients with ICH compared to other scoring systems (e.g., CONUT). Therefore, OPS and NPS might be better markers for predicting the outcomes of patients with spontaneous ICH.

Another important indicator for patients’ inflammatory immune status is CRP levels. However, the relationship between CRP and ICH is unclear. A previous study indicated that a raised CRP on admission was independently associated with an unfavorable outcome in patients with ICH [37]. Other studies have shown that a higher CRP level on hospital admission was consistently associated with hematoma growth [38]. However, some studies have shown that CRP concentrations were not associated with ICH [39, 40]. In our study, we found that CRP was higher in the group of ICH patients with a poor outcome; however, this was not statistically significant. Further research is needed to investigate this possibility further, particularly in the form of prospective, controlled and double-blinded studies.

It has previously been shown that changes in nutritional status do not immediately translate into weight loss; nevertheless, blood biochemical values may alter during the acute phase after ICH [41]. Albumin is synthesized by hepatocytes and is widely considered to be an marker for nutritional status. Low serum albumin levels might be independent predictors for a poor prognosis in patients with ICH [41]. Another study reported that hypoalbuminemia is associated with a systemic inflammatory response [42]. Our results demonstrated that patients in an unfavorable outcome group had low serum levels of albumin. Serum cholesterol level, another nutritional indicator, is also associated with malnutrition during the acute phase response [43]. A previous study showed that an increase in the levels of inflammatory cytokines, including interleukin-1 (IL-1) and tumor necrosis factor (TNF) can influence the metabolism of cholesterol and lead to hypocholesterolemia [44]. Moreover, a relationship between hypocholesterolemia and mortality has been identified in critically ill patients following surgery [45]. Another study showed that lower serum levels of cholesterol during the acute stage of ischemic stroke is an independent predictor of outcome [12]. Our present findings also suggested that total cholesterol levels in the poor outcome group were lower than those in the group of patients with favorable outcomes.

Based on the results of this study, it is reasonable to hypothesize that OPS and NPS scores can be used to guide the stratification of risk in ICH patients with poor prognosis and provide personalized treatment during the early stages of ICH. In theearly stages of ICH, an increase in enteral nutrition calorific intake is associated with improved neurological outcome at hospital discharge [13]. Thus, at the time of admission, it is important to evaluate the severity of patients with ICH so as to make rational treatment decisions and take appropriate actions to minimize the decline in the nutritional and immune status of ICH patients.

There are several limitations in our study. Firstly, this study was conducted in a single center; therefore selection bias was inevitable. Secondly, the study lacks a prospective and multicenter design, and the threshold value obtained from the total sample size may be biased. The cut-off values may vary from different regions and populations. Thirdly, the telephone follow-up after 6 months was only subjective evaluation, lacking objective measures. Fourthly, we only considered whether surgery was performed and did not take into account the surgical approaches or operation methods, which may be relevant to patient outcomes. Finally, we only included serum indicators at admission and not at 6-month follow-up.

## Conclusions

In patients with spontaneous ICH, those with a higher OPS and a higher NPS score on admission are likely to have a worse outcome at 6 months post-discharge. Our findings suggest that OPS and NPS may be valuable markers for predicting the outcomes of patients with spontaneous ICH.

## Data Availability

The data used and/or analyzed during the current study are available from the corresponding author upon reasonable request.
